# Impact of natural-resource dependence on foreign contracting projects of China: A spatial panel threshold approach

**DOI:** 10.1371/journal.pone.0234057

**Published:** 2020-06-11

**Authors:** Yu Sun, Huaping Sun, Lizhen Chen, Farhad Taghizadeh-Hesary, Guimei Zhao

**Affiliations:** 1 School of Finance and Economics, Jiangsu University, Zhenjiang, P.R. China; 2 Tokai University, Tokyo, Japan; School of Economics, Xiamen University, China, CHINA

## Abstract

The proposal of the “Belt and Road” initiative has had a positive and far-reaching impact on the economic and social development of countries and regions along the route and has provided good opportunities and conditions for the development of China’s foreign contracted projects. In the present study, in view of the heterogeneous characteristics and spatial correlation of countries along the Belt and Road, panel data of 46 contracted projects in China along the Belt and Road from 2008 to 2017 were used to empirically study the spatial characteristics of resource heterogeneity and outsourcing projects in the host country from the perspectives of spatial correlation and spatial heterogeneity. The results indicated that China had significant spatial agglomeration effects, natural restraining effects, and spatial spillover effects on the contracting projects along the Belt and Road, and the marginal impact in low-income countries exhibited a “broken line” relationship. Corresponding suggestions were provided for Chinese enterprises contracting projects involving Belt and Road countries. The databases of BRI need to be established, and ensure green investment efficiency.

## 1. Introduction

In 2018, global economic growth, shrinkage of the international engineering market, and increased economic friction produced complex and profound changes in the world political and economic structure. In this context, the “Belt and Road” initiative has become China’s preferred strategy to guide bilateral and multilateral international cooperation. With the implementation of the “Belt and Road” strategy, the momentum of China’s foreign contracted engineering business has accelerated. In 2018, the overall scale of China’s foreign contracted engineering business increased steadily, with a turnover of US$ 169.04 billion, i.e., an annual increase of 0.3%. The turnover of contracted projects in the countries along the Belt and Road reached US$ 89.33 billion, accounting for 52.8% of the total of contracted projects during the period, up to 4.4% annually. The first large-scale energy project of the China-Brazil Economic Corridor was put into operation at the Sahiwal Power Station, the first phase of the Sino-Thai Railway was started, and the Sino-Myanmar crude oil pipeline was officially put into use, as was the Port of Colombo in the Maritime Silk Road and the Greek city of Piraeus. The construction of projects, such as port sports (a large-scale key cooperative project undertaken by Chinese enterprises) is remarkable. These projects have not only played a leading role in industry but also promoted the economic and social development of countries along the line. This has led to the sustainable development of China’s corporate business and enhanced China’s international influence.

The country’s contracted projects along the Belt and Road have wide coverage, involve many countries, and make different contributions to economic development. China’s investments in contracting projects in the countries along the route are over-concentrated in ASEAN, West Asian, and Central and Eastern European countries. The spatial spillover effects have not been completely considered, and the equilibrium pattern of “multi-point, face-to-face synergy” has not been formed. This raises certain questions, for example, what factors affect China’s contracted projects along the Belt and Road countries? Location factors include “hard” factors and “soft” factors. The former refers mainly to natural resources, such as oil and mineral resources. In this study, the spatial perspective was used to analyze the factors influencing China’s foreign contracted projects along the Belt and Road countries, with consideration of the heterogeneity factors of the host countries. The objective was to provide advice for Chinese enterprises involved in contracted projects overseas for increasing the return on China’s foreign investment.

Foreign contracting projects involve transaction cooperation, including capital, technology, management, and labor services. This is related to (but obviously different from) foreign direct investment (FDI), scientific and technological cooperation, information and management cooperation, development assistance, and other forms of international economic cooperation. Friedman [1] presented research conclusions on international project contracting for the first time. Casson [2] used internalization theory to study the construction industry in terms of international engineering contracting. Enderwick [3] investigated the characteristics of the construction industry in foreign project contracting and the behaviors of the contracting enterprises and applied the international production compromise theory to the analysis of the specific engineering project practice.

Relatively few academic studies have been performed on foreign contracting projects involving countries along the Belt and Road, and few studies have focused on the financing of infrastructure construction along the Belt and Road. Pollio [4] analyzed the relationship between contracting enterprises, financial institutions, and the government from the perspective of the participants in project contracting and presented views on the financing problems in project contracting. Low and Jiang [5] analyzed 35 representative contracting enterprises and concluded that the market development ability and business level of China’s foreign contracting must be improved. Robert et al. [6], Wang [7] summarized the factors that should be considered when large engineering contractors enter the market in different regions. Sun et al. [8] discussed low-carbon financial risk factor correlation in the belt and road PPP project.

According to the characteristics of the location distribution of the Belt and Road contracted projects, different enterprises are affected by different factors when making investment location choices. Some scholars believe that the market size of the host country can significantly influence the location choice of outward FDI (OFDI) for enterprises. To transfer excess production capacity, Chinese enterprises are likely to seek a market in developing countries that are at a similar stage of development to China [9,10]. With the development of China’s domestic economy and foreign trade, China’s resource development enterprises pay more attention to the mineral resources and energy situation of the host country [11]. With an increase in the market size the resource utilization efficiency and opportunity to develop a scale economy increase, and there are better investment opportunities for foreign investors. Buckley [12], Chen et al. [13] used an empirical panel regression model to investigate the factors affecting China’s OFDI and found that China’s foreign investment shows for the market and find the motivation of resource. However, the drivers in developed and developing countries are not identical. The Chinese capital in developed countries exhibits clustering, whereas that in developing country is scattered. Wang et al. [14] studied data for 56 countries in Asia, Africa, and Latin America and found through empirical regression that the economic growth, market size, energy, labor, and other factors of the host country had a significant positive correlation with China’s foreign contracted project investment. However, the infrastructure of the host country had no significant impact on China’s foreign contracted project investment. Scholars [15,16,17] have used the classical gravity model to select countries along the “Silk Road Economic Belt” as samples; added variables, such as the urban population ratio, interest rate, and R&D personnel; and performed empirical regression analysis using the panel data model. Their results indicated that the participation of the host country in a trade agreement, the proportion of urbanization, and the level of urbanization are all important factors affecting the entry of China’s contracted projects. Other scholars [18,19] consider that the economic scale, labor force scale, and investment environment of the host country are significantly positively correlated with the scale of China’s outbound investment and that among these factors, improvement of the investment environment plays the greatest role in promoting China’s investment.

Researchers have investigated the effects of foreign contracted projects, including Zhang [20], who examined the development process and current situation of China’s overseas contracting business. Cai et al. [21] conducted an in-depth investigation of the influence of China’s foreign contracting projects on the OFDI, performed an empirical study based on a panel data model, and concluded that China’s overseas engineering business contracting plays a significant role in promoting OFDI. As the market size increases, the resource utilization efficiency and opportunities for developing economies of scale and scope increase, and more investment opportunities exist for foreign investors [22,23].

After the spatial agglomeration of investment activities reaches a certain scale, spatial spillover can occur. Ayhan et al. [24] found that the spillover effect of FDI not only occurs in the host country but also might result in a cross-regional diffusion phenomenon owing to spatial correlation, and competition and imitation of adjacent markets. Studies have verified that the economic effect of FDI in the host country can improve the productivity of the contracting country through spillover to surrounding countries or regions, having a positive spatial spillover effect [25]. However, studies have also revealed a negative spillover effect [26]. Aitken et al. [27] called this negative effect of foreign capital flows on local productivity “market theft.” Ma and Liu [28] used the spatial durbin model (SDM) to test the effect of China’s OFDI to third-world countries and observed a significant crowding-out effect. Many other scholars have studied the technological or institutional factors in the green transformation of resource economy [29–33]. Therefore, it is important to select appropriate measurement tools and variables for different research objects.

As indicated by the literature, most of the studies on foreign contracted projects have focused on data description and individual case analysis, lacking systematic theoretical analysis and detailed empirical testing of data. What is the impact of the host country’s natural resources on China’s overseas contracting projects along the “Belt and Road”? In this study, to address these questions and supplement the existing research, a spatial model was used to investigate the heterogeneous performance of factors influencing foreign contracting projects in the countries along the “Belt and Road”.

## 2. Materials and methods

### 2.1. Spatial panel econometric model

The most widely used spatial econometric models are the spatial autocorrelation model, spatial error model (SEM), and SDM. The SDM is a general form of the spatial lag model and SEM and it expressed as follows:

$$y_{\mathrm{it}}=c+\rho \sum_{j=1}^{n}W_{\mathrm{ij}}y_{\mathrm{it}}+\alpha X_{\mathrm{it}}+\sum_{j=1}^{n}W_{\mathrm{ij}}X_{\mathrm{it}}\gamma +\mu_{i}+\lambda_{t}+\varepsilon_{\mathrm{it}}$$ (1)

where *y*_it_ is the dependent variable, *X*_it_ is the independent variable, *α* is the independent variable coefficient, *c* is a constant, *ρ* is the spatial autoregressive coefficient of the dependent variable, *γ* is the spatial lag coefficient of the independent variable, and *μ*_i_ and *λ_t_* represent the spatial effect and the time effect, respectively. The residual term is *ε*_it_, and *W*_ij_ is a spatial weight matrix that indicates the degree of correlation and mutual influence between the spatial elements. *αX*_it_ indicates the influence of the independent variable of the region on the dependent variable, and *W*_ij_*X*_it_ indicates the influence of the independent variable of the region on the dependent variable in the neighboring region, that is, the spatial spillover effect.

### 2.2. Spatial autocorrelation test

The results of spatial regression estimation have research significance only if there is a significant spatial correlation. Therefore, the Global Moran’s I was used to verify the spatial correlation of the entire spatial sequence.

$$I=\frac{\sum_{i=1}^{n}\sum_{j=1}^{n}W_{ij}\left(X_{i}-\bar{X}\right)\left(X_{j}-\bar{X}\right)}{\sum_{l=1}^{n}{\left(X_{i}-\bar{X}\right)}^{2}}$$ (2)

### 2.3. Variable selection and description

In this study, China’s foreign contracted projects along the “Belt and Road” are selected as the explanatory variables. According to the availability and continuity of the sample data, 46 member countries along the “Belt and Road” in 2008–2017 are selected as the samples. In combination with the theory of comparative advantage, FDI should be selected from industries that are about to lose their comparative advantage in their own country but have a comparative advantage in the host country. According to the international production eclectic theory, under the market incompleteness assumption, an enterprise’s direct international investment is determined by the following three factors: the location advantage, internalization advantage, and ownership advantage of the enterprise. China’s Belt and Road strategy is based on this theory. The new economic geography has intensified the specific location elements, transportation costs, and imperfect competition market and scale returns into the central–peripheral model, which explains the dynamic formation process of the agglomeration economy.

According to the foregoing theory, natural-resource endowment is selected as the core variable, and the market scale of the host country, labor cost, openness of the host country, and infrastructure index of the host country are selected as the control variables. See Table 1 for details of the variables.

**Table 1:** Description of related variables and data sources.

Variable	Variable description	Data source
FCP	China’s turnover of foreign contracted projects along the Belt and Road countries	http://data.stats.gov.cn/easyquery.htm?cn=C01
Resource	The natural-resource endowment of the host country is the ratio of the total natural-resource rent to the gross domestic product (GDP).	https://data.worldbank.org.cn/indicator/NY.GDP.TOTL.RT.ZS?view=chart
Open	The degree of openness of the host country is calculated from the proportion of the host calculated as the ratio of the host country’s exports to the GDP.	https://data.worldbank.org.cn/indicator/NE.EXP.GNFS.ZS?view=chart
Infrast	Infrastructure construction, expressed in terms of the number of mobile devices per 100 people	https://data.worldbank.org.cn/indicator/IT.MLT.MAIN.P2?view=chart
Pop	Population of the host country	https://data.worldbank.org.cn/indicator/SP.POP.TOTL?view=chart
GDP	Real GDP of the host country	https://data.worldbank.org.cn/indicator/NY.GDP.MKTP.CD?view=chart

## 3. Results

### 3.1. Spatial effect test

To test the stability of the spatial panel model, we used four different spatial weight matrices: a spatial adjacent matrix, a geographic distance matrix, an economic distance matrix, and an economic geographic distance nesting matrix. According to the set spatial weights, the global Moran’s index of China’s national project contracting along the Belt and Road was calculated. The corresponding index and test results are presented in Table 2.

**Table 2:** China’s global spatial correlation of FCP along the Belt and Road.

Year	Spatial adjacent matrix		Geographic distance matrix		Economic distance matrix		Geographic economic nesting matrix	
	I	p-value	I	p-value	I	p-value	I	p-value
2008	0.302	0.005	0.273	0.000	0.148	0.009	0.149	0.009
2009	0.383	0.001	0.351	0.000	0.164	0.005	0.162	0.005
2010	0.428	0.000	0.289	0.000	0.132	0.017	0.133	0.016
2011	0.492	0.000	0.349	0.000	0.189	0.002	0.190	0.002
2012	0.460	0.000	0.299	0.000	0.183	0.002	0.182	0.002
2013	0.334	0.002	0.243	0.002	0.149	0.009	0.149	0.009
2014	0.143	0.093	0.133	0.040	0.090	0.059	0.089	0.061
2015	0.202	0.039	0.201	0.007	0.085	0.070	0.084	0.073
2016	0.294	0.006	0.247	0.001	0.048	0.166	0.048	0.166
2017	0.428	0.000	0.383	0.000	0.063	0.122	0.063	0.119

According to the spatial correlation test results in Table 2, the positive correlation of the external engineering contract space is significant only for the geographic distance matrix. Therefore, the geographic distance matrix is used as a weight matrix for further analysis.

### 3.2. Applicability test of the space spatial model

Owing to the large number of spatial panel models, the spatial panel model must be further tested, and an appropriate model must be selected to avoid deviations in the model settings. The main test steps are as follows. First, the Lagrange multiplier (LM) is used to test the autocorrelation between the dependent variable and the spatial error of the spatial lag, and the robust LM statistic is used to check for the existence of spatial interaction effect. We set either the spatial fixed effect or the time fixed effect to select the SEM and the spatial lag model. Second, the Wald and likelihood ratio (LR) statistics are used to determine whether the SDM will become a spatial error or a spatial lag model. Furthermore, the Hausman statistic is used to select fixed effects and random effects to improve the panel effects. Finally, the LR test results are used to select the time fixed-effect model, spatial fixed-effect model, and space–time double fixed-effect model. The results of the model test are presented in Tables 3–5.

**Table 3:** Applicability LM test of the spatial model.

Panel effect	No effect	Space fixed effect	Time fixed effect	Double fixed effect
LM_lag	0.3882	1.6036	0.0084	3.8941**
LM_lag(robust)	18.4259***	7.4091***	13.9581***	2.5830*
LM_error	2.4820*	0.5872	4.4109**	5.2614**
LM_error(robust)	20.5197***	6.3927***	18.3606***	3.9503**

*, **, and *** indicate significance at the 0.1, 0.05, and 0.01 levels, respectively.

**Table 4:** Spatial panel model Wald test results.

Test type	Wald_spatial_lag	LR_spatial_lag	Wald_spatial_error	LR_spatial_error
Time–space fixed effect	42.0531***	40.4083***	40.1439***	38.5044***
Spatial random, time fixed effect	60.9702***	55.0583***	58.3884***	41.4798***

*, **, and *** indicate significance levels of 0.1, 0.05, and 0.01, respectively.

**Table 5:** Spatial panel model Hausman test and LR test results.

Test type		Statistics
Hausman test		55.1853***
LR test	Spatial fixed effect	486.9380***
	Time fixed effect	44.9464***

*, **, and *** indicate significance at the 0.1, 0.05, and 0.01 levels, respectively.

To further validate the applicability of the spatial model, the LM-lag and LM-err statistics and robust LM-lag and LM-err statistics are used for testing. As shown in Table 3, under the four effects, the level of statistical significance of the SEM is better than that of the spatial lag model. Therefore, the SEM is selected for model regression. To ensure that the estimation results of the spatial model are robust, we continue to use the LR test. As indicated by Tables 4 and 5, in both the Wald test and the LR test, the null hypotheses are rejected. The results indicate that the SDM cannot be reduced to either the spatial lag model or the SEM. Therefore, the SDM should be used in the construction of the model. Finally, the Hausman test is used to judge the choice of either a fixed or random effect. The test results indicate that the null hypothesis is rejected at the 1% significance level and that the fixed-effect model is better than the random-effect model. According to the LR test results, for the time fixed-effect and spatial fixed-effect models, the original hypothesis is significantly rejected. Thus, the empirical test should select the SDM with a two-way fixed effect for the next regression.

### 3.3. Empirical results

To eliminate the effects of heteroscedasticity, the variable GDP is treated logarithmically. Because of the spatial lag term of the independent variable in the SDM, the above estimated coefficient does not directly reflect the marginal effect of the independent variable on the dependent variable but rather reflects the level of significance and direction of action (and thus the independent variable). The effects can be classified as direct or indirect.

As indicated by Table 6, except for the infrastructure construction of economic host countries, other variables are significant. The direct and fixed effects of the host country’s resource endowment both exhibit significantly negative values, indicating that a higher proportion of natural-resource rent in the GDP of the host country corresponds to a smaller amount of China’s project contracting in the country. The more obvious resource-seeking motivation exists in China’s project contracting in countries along the Belt and Road, and the high cost of natural resources in the host country hinders China’s investment. Although the growth of China’s energy-intensive manufacturing industry has slowed in recent years, China’s dependence on natural-resource products will remain at a high level. Factors such as the market openness, labor costs, and market size of the host country pass the significance test at the 5% level, whereas spatial spillover is not significant, indicating that the effects of these factors are mainly limited to the local region and that the spatial spillover effect of other regions is small. This suggests that a larger market size of the host country corresponds to a larger direct investment from China in the country. A larger market scale of the countries along the line corresponds to a larger demand for products of Chinese enterprises; thus, in countries with different development levels along the line, China’s foreign project contracting has obvious market-seeking motivation. The coefficient of the labor cost variable is significantly negative, indicating that the labor cost of countries along the line has a significant negative impact on China’s foreign project contracting. A higher labor cost corresponds to a smaller direct investment from China. With the development of China’s economic level, China’s labor advantage has gradually disappeared, and the labor supply has become a bottleneck. Most of the countries along the Belt and Road are developing countries with low labor costs, which easily attract China’s cost-driven investment. The host country’s export effect substitutes for China’s investment, and the combination of natural resources has a negative impact on China’s enterprise investment, which effectively counters the international community’s criticism of China’s “resource plunder” investment.

**Table 6:** Spatial econometric results of the SDM.

Variable	Fixed effect	Direct effect	Indirect effect	Total effect
Resource	–26.09158**	–25.62047**	76.4473**	50.82683
	(–2.38)	(–2.24)	(2.37)	(1.56)
Open	–18.9498***	–19.02261***	–0.72614	–19.74875***
	(–3.59)	(–3.75)	(–0.18)	(–3.01)
Infrast	–1.682167	–1.714129	–0.1516159	–1.865745
	(–0.72)	(–0.75)	(–0.24)	(–0.74)
Pop	-0.0253851***	–0.0249191***	–0.001088	–0.026007***
	(–5.41)	(–5.53)	(–0.21)	(-3.59)
LnGDP	1581.425***	1572.883***	60.22585	1633.109***
	(6.56)	(6.69)	(0.19)	(4.31)

*, **, and *** indicate significance at the 0.1, 0.05, and 0.01 levels, respectively.

## 4. Discussion

According to the foregoing analysis, the impact of the host country’s natural-resource endowment on China’s foreign project contracting is not characterized by a simple linear relationship. Moreover, “the Belt and Road” has a large economic volume, and there are significant differences in the levels of economic development of the countries along the Belt and Road. Therefore, we perform an analysis to determine whether there is a threshold effect in the contribution of infrastructure to economic growth in different regions from 2008 to 2017. We use Hansen’s threshold panel model:

$$FCP_{it}=\beta_{0}+\beta_{1}resource(x_{it}\leq \gamma )+\beta_{2}resource(x_{it}>\gamma )+\beta_{j}x_{jit}+\varphi_{i}+\varepsilon_{it}$$ (3)

The test process of panel threshold regression includes two main steps: 1) determine whether the threshold effect exists, and if it exists, estimate the threshold value; 2) test the significance level of the threshold effect. First, according to an overall analysis of the 46 countries along the Belt and Road and the World Bank’s economic levels, the countries along the line are divided into two groups according to their income level. High- and middle-income countries are classified as high-income groups, and low- and medium-income countries are classified as low-income groups. In the analysis, the factors influencing the natural-resource endowment for China’s foreign contracting projects at different development levels are compared.

As shown in Table 7, the single threshold of natural-resource endowment passed the significance test, indicating that the natural-resource endowment has a single-threshold effect on China’s foreign engineering contracting along the Belt and Road. For 2008–2017, the natural-resource endowment threshold effect for middle- and high-income countries is insignificant; indicating that the natural-resource endowment of the middle- and high-income countries along the Belt and Road is relatively stable to China’s external contracting. Additionally, the threshold of natural resources for low-income countries is significant, indicating that the natural-resource endowment of low-income countries and the impact of China’s foreign project contracting undergo a sudden change. Perhaps, with the continuous improvement of China’s technology level, scale effect, management quality, and other factors, the impact of continuous investment in natural resources on foreign project contracting will decrease gradually; thus, the mutation phenomenon is likely to be a transition from a high marginal return to a low marginal return.

**Table 7:** Panel threshold effect test and threshold estimation results.

Threshold variable	Hypothesis test	bootstrapF	Critical value of significance level		
			90%	95%	99%
Resource	HO: no threshold value; H1: one threshold value	34.01*	32.886	41.8052	64.8927
Resource(HIGH)	HO: no threshold value; H1: one threshold value	10.63	27.7128	36.5301	67.0479
Resource(LOW)	HO: no threshold value; H1: one threshold value	33.66*	25.7119	41.7883	65.7822

Bootstrap samples are taken 300 times; F-test statistics are used, and * indicates significance at the level of 0.1.

According to the test results for the threshold variables in Table 7, a single-threshold regression of a fixed-effect panel is conducted for those with one threshold value. The estimated threshold values and threshold variable parameters are presented in Table 8.

**Table 8:** Estimated threshold values and parameters.

Variable	Estimated Value	T Value	P Value
Resource < 3.1771	–401.1882**	–2.23	0.031
Resource > 3.1771	–44.11979**	–2.36	0.023
Resource low < 2.8628	–590.59*	–2.00	0.061
Resource low > 2.8628	–50.1629	–1.48	0.157

*, **, and *** indicate significance at the 0.1, 0.05, and 0.01 levels, respectively.

Table 8 presents the estimated threshold value of natural-resource endowment and threshold variable parameters. The threshold value reflects the critical value of sudden change, and the parameters reflect the direction and degree of influence of the natural-resource endowment on China’s foreign project contracting.

## 5. Conclusions

China’s contracting space pattern along the “Belt and Road” project was analyzed, and the SDM was employed to empirically examine the influencing factors and decompose the direct and indirect effects. The following conclusions are drawn:

1. The spatial correlation of China’s contracted projects in 46 countries along the Belt and Road was tested. With regard to the global Moran’s index, the contracting projects exhibited a significant positive spatial correlation and a gradual upward trend, indicating its existence of spatial effect. According to the Moran’s index results, China has a significant spatial agglomeration effect on national contracting projects along the Belt and Road.
2. The results for the resource endowment of the host country indicated that there is a resource-seeking motivation in China’s engineering contracting in the countries along the Belt and Road and that the high cost of natural resources in the host country discourages China from contracting local projects. A higher level of economic development of the host country corresponds to a larger potential consumer market and makes it easier for Chinese-invested enterprises to obtain raw materials and labor and enter consumer markets. Additionally, it is easier for these enterprises to utilize various resources and adjust production according to changes in consumer demand for increasing their income. Labor cost has a restraining effect on China’s project contracting in the host country; a higher labor cost corresponds to less project contracting with China in the local area.
3. The marginal influence of the natural-resource endowment of the host country on China’s foreign project contracting exhibited a downward trend. There was a significant threshold effect. With an increase in the threshold value, the possibility of parameter estimation of the threshold variable decreased. This was more evident for lower-income countries. With the traditional policy incentives that drive investment growth via resource inputs no longer having stable benefits, policymakers are forced to change the traditional incremental approach and focus on improving investment management quality.

To enhance the spatial agglomeration effect and the spillover effect of China’s national project contracting along the Belt and Road, the global investment scale of the “Belt and Road” should be expanded, and the spatial equilibrium strategic layout of “multi-point and surface-wide coordination” should be realized. Thus, the following are our recommendations.

First, spatial agglomeration and spatial spillover effects should be employed to realize multi-point and face-to-face coordination. It is necessary to utilize the spatial agglomeration effect to transition from a “development space agglomeration point” to “regional coordinated development.” China’s project contracting for countries along the Belt and Road is related not only to the two main bodies (China and the host country) but also to the neighboring countries of the host country. Therefore, in the process of contracting foreign projects in China, we should not only focus on bilateral relations but also establish a cooperative win–win mechanism and strive to achieve regional coordination and common development.

Second, the level of infrastructure development in countries along the Belt and Road also matters. Infrastructure construction is the core of the Belt and Road strategy. Only by connecting the facilities can we appropriately match the rules and regulations and the exchange of personnel. With the implementation of the Belt and Road strategy, China’s investment in infrastructure construction along the route has increased; however, further attention should be paid to infrastructure construction in low-income and less developed countries and to enhancing the infrastructure for investment in local communication facilities.

Third, the advantages of each country should be used to develop differentiated regional investment strategies. The environmental development of the countries along the Belt and Road, such as economic and social development, market perfection, and infrastructure construction, is uneven. The risks and obstacles faced by Chinese enterprises are also different. Thus, China should formulate different investment strategies for countries with different development levels and environments along the Belt and Road.

Finally, to avoid political risks, a Belt and Road risk information platform should be constructed, and bilateral investment agreements should be signed. The empirical results of this study indicate that the host country’s market and openness, along with other factors, significantly affect China’s foreign engineering contracting projects. Thus, Chinese enterprises’ investment in countries along the Belt and Road may face many risks in the host countries. Most developing countries along the Belt and Road are in transition as emerging markets. There are problems, such as imperfect market mechanisms and imperfect social and political systems. The Belt and Road project is still at a relatively early stage, and China has not had a large amount of experience with foreign investment enterprises. Therefore, in the future we should systematically evaluate the risk categories of the Belt and Road countries, establish a database of Chinese enterprises that are “going global” along the Belt and Road, and provide scientific guidance and suggestions for Chinese enterprises to “go global”.
